# The Association between the Pulmonary Arterial Obstruction Index and Atrial Size in Patients with Acute Pulmonary Embolism

**DOI:** 10.1155/2019/6025931

**Published:** 2019-06-02

**Authors:** Taraneh Faghihi Langroudi, Maryam Sheikh, Mohammadreza Naderian, Morteza Sanei Taheri, Amir Ashraf-ganjouei, Isa Khaheshi

**Affiliations:** ^1^Radiology Department, Modarres Hospital, Shahid Beheshti University of Medical Sciences, Tehran, Iran; ^2^Non-Communicable Diseases Research Center, Endocrinology and Metabolism Population Sciences Institute, Tehran University of Medical Sciences, Tehran, Iran; ^3^Cardiac Outcome Research and Education (CORE), Universal Scientific Education and Research Network (USERN), Tehran, Iran; ^4^Students' Scientific Research Center (SSRC), Tehran University of Medical Sciences, Tehran, Iran; ^5^Cardiovascular Research Center, Shahid Beheshti University of Medical Sciences, Tehran, Iran

## Abstract

**Purpose:**

Pulmonary embolism (PE) is a common and potentially fatal form of venous thromboembolism. The aim of this study is to investigate the association between the pulmonary arterial obstruction index and atrial size in patients with acute pulmonary embolism.

**Basic Procedure:**

The study consisted of 86 patients with clinical symptoms of PE. Out of 86 individuals, 50 patients were diagnosed with PE and considered as the patient group. The others were considered as the control group. All patients were scanned by a multidetector CT scanner. Using the radiology workstation, an expert radiologist calculated the left atrium (LA) and right atrium (RA) areas from planimetric measurements obtained from free-hand delineation of the atrial boarders using an electronic pen. Quantitative volumetric measurements of LA and RA were obtained from original axial images.

**Main Findings:**

There were 25 males and 25 females with PE, who had a mean age of 58 years. There was not a significant difference in the positive history of diabetes mellitus, hypertension, asthma, chronic obstructive pulmonary diseases, ischemic heart disease, and smoking between patients and control group. There was a significant negative correlation between almost all LA measurements and the PAOI. RA area and volume had the highest area under the curves for recognizing larger clot burden.

**Principal Conclusions:**

A higher clot load is associated with a smaller LA size and increased RA/LA ratios, measured with CTPA. Atrial measurements are correlated with POAI, and they could be used as sensitive parameters in predicting heart failure in patients with PE.

## 1. Introduction

Pulmonary embolism (PE) is a common and potentially fatal form of venous thromboembolism that causes 200,000-300,000 deaths annually in the United States, mostly occurring in patients with hemodynamically unstable status [1].

Patients with unrecognized acute PE have an estimated mortality rate of 30% [2]. However, the death rate can be reduced to 2-10% if PE is diagnosed at the right time [3]. Computed tomography pulmonary angiography (CTPA) is currently the modality of choice for the evaluation of acute pulmonary embolism [4]. CTPA has a specificity of 95% for identifying clots within distal pulmonary arteries. Moreover, using CTPA can help physicians in the diagnosis of underlying lung diseases [3]. CTPA can also assess clot burden, using scoring systems such as pulmonary artery obstruction index (PAOI) [5].

Acute PE increases the pressure of the pulmonary arterial system and right ventricle (RV) resulting in right ventricle dysfunction (RVD) [6]. RVD followed by PE may cause death generally within the first hour following admission to hospital [7]. Although echocardiography is currently the modality of choice for RVD diagnosis, CPTA can replace echocardiography, because of its ability to detect PE and RVD simultaneously [8]. Studies show that among patients with PE, the PAOI ratio can recognize patients with or without RVD [6, 9].

Determination of CTPA ability in the early detection of circulatory collapse in patients who have increased risk of acute right heart failure caused by PE is still going [10]. In current study, we have investigated the association between the pulmonary arterial obstruction index and atrial size in patients with acute pulmonary embolism.

## 2. Material and Methods

### 2.1. Study Participants

The study cohort consisted of 86 patients with clinical symptoms of PE who were referred to Shahid Modarres Hospital in Tehran from April 2015 to May 2017. Out of 86 individuals, 50 patients were diagnosed with PE on CTPA and were considered as the patient group. The other 36 patients who did not have any signs of PE on CPTA were considered as the control group. The ethical committee of Shahid Beheshti University of Medical Sciences approved this study and written informed consent was obtained from all participants.

### 2.2. CT Acquisition and Assessment

**Figure 1:**
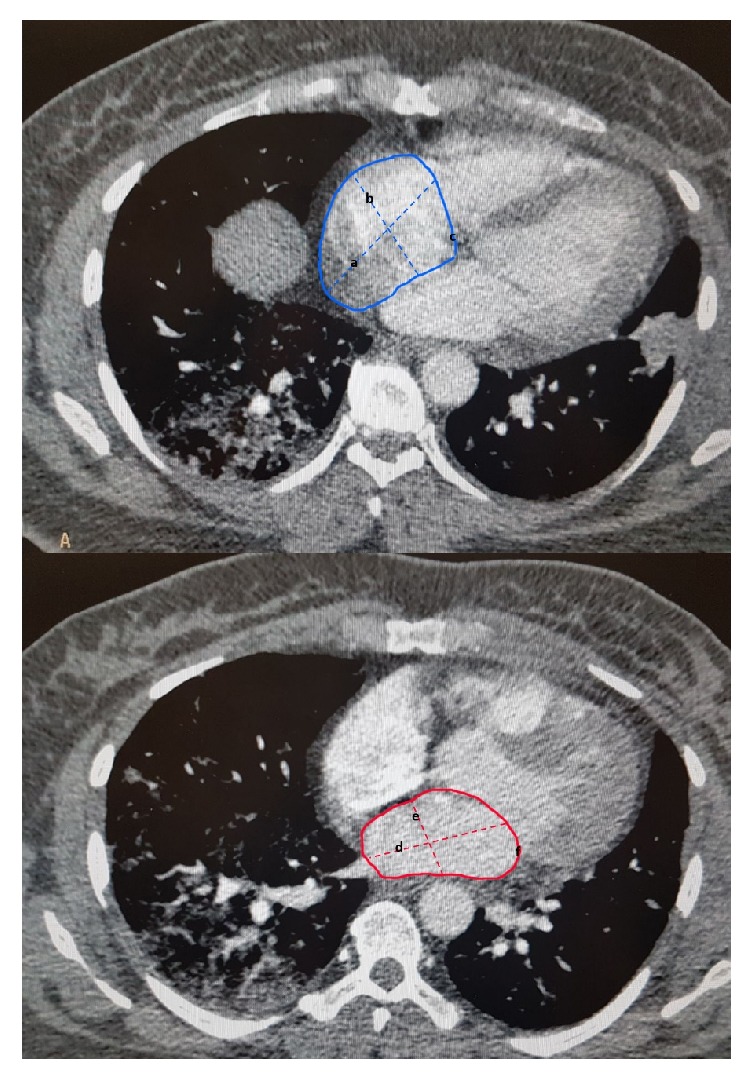
Radiological indices defined in computed tomography pulmonary angiography (CTPA). The figure, depicting a sample of computed tomography pulmonary angiography (CTPA) image and the indices defined for measuring different sizes. a, b, and c imply RA long diameter, short diameter, and area, respectively. d, e, and f imply LA long diameter, short diameter, and area, respectively.

All patients were scanned by a multidetector CT scanner (Brilliance 64, Philips medical system, Cleveland, OH, USA). Reconstructed slice thickness was 1.0 mm, with an increment of 0.5 mm. The PE protocol consisted of contrast injections of 50 mL of iodinated contrast material at a concentration of 320 mg iodine/ml (Visipaque, GE healthcare, Ireland, Cork, Ireland). All CT scans were taken at end-of-inspiration during a single breath-hold in a caudal-cranial direction. Using the Radiology workstation (extended brilliance workspace, Philips medical systems Nederlan B.V.), an expert radiologist calculated the left atrium (LA) and right atrium (RA) areas from planimetric measurements obtained from free-hand delineation of the atrial boarders using an electronic pen. Quantitative volumetric measurements of LA and RA were obtained from original axial images, with the protocol used by Aviram et al. [10] (Figure 1). The location of arterial clot presence and the degree of arterial obstruction were scored using the scoring system suggested by Qanadli et al. in this order, the CT obstruction index was described as (n. d) [n, value of the proximal clot location, equal to the number of segmental divisions arising distally; d, extent of obstruction scored as partial obstruction (value of 1) or total obstruction (value of 2)] [5].

### 2.3. Statistical Analysis

Demographic data were presented as the number of individuals for categorical variables, and as mean with standard deviation (SD) for continuous variables. Student's t-test and Pearson's *χ*^2^ were used to evaluate basic demographic and clinical information in case and control groups. Spearman's rank correlation coefficient was used to assess the correlation between PAOI and LA and RA measurements, as well as LA to RA ratios. Furthermore, to analyze the performance of classification schemes for the different parameters and to compare their ability for identifying higher clot loads, we used a receiver operating characteristic (ROC) curve analysis. Statistical analysis was done using the statistical package SPSS version 22 (IBM Corp., Armonk, N.Y., USA) and* P values* under 0.05 were considered to have statistical significance.

## 3. Results

There were 25 males and 25 females with PE, who had a mean age of 58 years (SD of 17.93). The control group included 20 males and 16 females, who had a mean age of 55 years (SD of 17.56). The age difference between the two groups was not significant (*P value* of 0.42). The baseline characteristics of the study population are shown in Table 1. There was not a significant difference in the positive history of diabetes mellitus (DM), hypertension (HTN), asthma, chronic obstructive pulmonary diseases (COPD), ischemic heart disease (IHD) and smoking between patients and control group.

Table 2 shows the correlation between the POAI and radiologic parameters of left and right atrium (LA and RA, respectively) on CTPA. There was a significant negative correlation between almost all LA measurements and the PAOI. Regarding RA, only its short diameter was positively correlated with the PAOI. Consequently, RA/LA Short Diameter, RA/LA Area, and RA/LA Volume ratios were positively correlated with the PAOI. It is noteworthy to mention that in the group of patients, the minimum and maximum of PAOI were 1 and 30, respectively, while mean and standard deviation were 10.32 and 8.45, respectively.

Table 3 shows the results of the ROC curve analysis of the PAOI and different RA and LA measurements. We analyzed different cut-offs of the atrial parameters to recognize patients with a large clot burden. RA area and volume had the highest area under the curves. In addition, we had reported cut-offs that achieve 90% sensitivity and the corresponding specificity for these parameters in Table 3.

## 4. Discussion

The main finding of our study was that a higher clot load is associated with a smaller LA size and increased RA/LA ratios, measured with CTPA. Although RA long diameter, area, and volume were significantly higher in patients in comparison to control group, only an association between a higher clot load and RA short diameter was observed. On the other hand, ROC curve analysis showed that RA area and volume were capable of identifying patients with a large clot burden. Our study suggests that assessing LA and RA dimensions in patients with PE could give the physician some valuable information about the clot load and serve as another prognostic factor for PE.

Pulmonary embolism is one of the fatal conditions of circulatory system. Two series of events may contribute to PE mortality. First, massive PE results in pulmonary arterial hypertension, which subsequently causes pressure overload in right ventricle. This will decrease right heart output, resulting in right ventricular dysfunction and probably right ventricle failure [11]. Consequently, blood flow to left atrium and ventricle would be decreased. On the other hand, increased afterload in the right ventricle would cause dilation in this chamber. The dysfunctional and dilated right ventricle could displace the interventricular septum toward the left ventricle. The leftward shift of the septum impairs the left ventricular preload [7]. All of the mentioned circumstances could decrease cardiac output, resulting in hypotensive shock that could present with catastrophic clinical findings [12]. Studies had shown that even a remarkable proportion of PE patients could present with normal blood pressure while having right ventricular dysfunction [13]. These patients might develop PE-related hemodynamic instability, requiring aggressive therapy. Thus, investigating different variables with CTPA that predicts right and left heart failure could be lifesaving, especially in patients with a higher clot load.

Regarding the degree of vascular obstruction, Qanadli et al. proposed the CT obstruction index that was correlated with the previously described pulmonary angiography index, which could quantify the degree of obstruction [5]. They suggested that a CT obstruction index more than 40% could identify 90% of patients with dilated right ventricle, while an index less than 40% would be unlikely to be observed in a patient who is suffering from acute right ventricular dysfunction [5]. Mastora et al. described that CT severity index could quantitatively assess PE severity, suggesting an applicable scoring system for routine clinical practice [14].

Several studies have investigated pulmonary obstruction indices for their ability to select high-risk PE patients. Van der meer et al. described that both the PAOI and right ventricle (RV) to left ventricle (LV) ratio could be useful in predicting mortality in PE patients that are hemodynamically stable at the presentation [11]. Wu et al. also suggested that quantified CTPA clot burden analysis could have prominent predictive value on the clinical outcome of patients with PE [15]. On the other hand, Collomb et al., Araoz et al., Pech et al., and Ghaye et al. found that PE clot scores might be poor variables for predicting severe in-hospital morbidity and mortality [3, 16–18]. These contradictory findings could support the hypothesis that both basic cardiopulmonary state and PE clot size contribute to patients' outcome. Hence, there is not a definite variable with the highest predictive value for PE-related mortality in different populations.

Few studies with a limited number of patients have investigated the association between clot burden and left atrium size [12, 19]. A recent study by Aviram et al. proposed that LA and RA areas are associated with embolic extant, suggesting higher PAOI results in a larger RA and a smaller LA size [10]. However, our current study is the first to show that there is a correlation between almost all LA measurements and the PAOI in a number of PE patients. These findings confirm that assessing LA and RA dimensions could become an applicable tool for stratifying PE patients in daily clinical practice.

Studies had shown that although echocardiography should not be used as a routine modality for diagnosing PE, it can be useful for risk assessment in PE patients, especially those who may have poor prognosis due to RVD [20]. Lodato et al. investigated quantitative indices using echocardiography and proposed that RV/LV ratio has decent accuracy for diagnosis of PE [21]. Lim et al. compared the accuracy of echocardiography and CT in detecting of RVD in patients with acute PE [22]. Out of 14 patients with massive pulmonary embolism, echocardiography had detected RVD in 12 individuals, while CT had correctly identified 11 of 12 patients, resulting in a sensitivity of 91.6% and a specificity of 100% [22]. Moreover, He et al. evaluated qualitative assessment of qualitative assessment of RVD in patients with PE, on CT in comparison to echocardiography. CT had a sensitivity and specificity of 81% and 47%, respectively [23]. Since ventricular wall is significantly thicker than atrial wall, the effect of arterial obstruction on atrial size might be more noticeable. Our findings also suggest that RA area could be a sensitive parameter for the diagnosis of massive PE.

ECG-gated scanning technique was not used in our CT protocol. It is a lack for measurement of cardiac chambers. Also, echocardiography is better method for measurement of cardiac chambers. But in our study, correlation analysis between echocardiography and computed tomography measurements was not used. This point should be mentioned as a limitation of our study and recommended for future studies to be considered.

Furthermore, retrospective type of our study and small sample size are the other limitations of our study. Future investigations with large sample size can reveal new and detailed results.

In conclusion, we had investigated the association between pulmonary arterial obstruction index and atrial size in patients with acute pulmonary embolism. We found that atrial measurements are correlated with POAI, and they could be used as sensitive parameters in predicting heart failure in patients with PE. Further studies are needed to confirm this hypothesis.

## Figures and Tables

**Table 1:** Basic demographic and clinical information in case and control groups.

	Patients (N = 50)	Controls (N = 36)	P value
	Mean (SD), Median (IQR), N (%)	Mean (SD), Median (IQR), N (%)	
Male, n	25 (50)	20 (55.6)	0.61
Age, year	58.30 (17.93)	55.14 (17.65)	
Positive History of DM, n	11 (22)	3 (8.3)	0.09
Positive History of HTN, n	18 (36)	9 (25)	0.28
Positive History of Asthma, n	1 (2)	4 (11)	0.07
Positive History of COPD, n	2 (4)	4 (11.1)	0.20
Positive History of IHD, n	6 (12.2)	2 (5.6)	0.30
Positive History of Smoking, n	13 (26.7)	10 (27.8)	0.85
Hospital mortality, n	3 (6)	2 (5.6)	0.93

Reflux Grade, n	2 (1.44)	1.69 (1.49)	0.08

LA Long Diameter, cm	6.39 (1.10)	5.94 (1.16)	0.07
LA Short Diameter, cm	4.06 (0.98)	3.54 (0.87)	**0.01**
LA Height, cm	5.07 (0.99)	5.23 (0.81)	0.42
LA Area, cm^2^	20.95 (7.02)	17.75 (7.60)	**0.04**
LA Volume, cm^3^	69.28 (36.80)	58.52 (32.36)	0.16

RA Long Diameter, cm	5.13 (1.00)	4.40 (1.01)	**0.01**
RA Short Diameter, cm	5.21 (0.87)	4.87 (0.83)	0.07
RA Height, cm	7.47 (1.62)	7.11 (1.16)	0.25
RA Area, cm^2^	21.98 (6.48)	17.35 (6.08)	**<0.01**
RA Volume, cm^3^	102.84 (45.60)	78.38 (34.28)	**<0.01**

*Abbreviation*: DM: diabetes mellitus; HTN: hypertension; IQR: interquartile range; LA: left atrium; RA: right atrium; SD: standard deviation.

**Table 2:** Correlation between PAOI and radiologic parameters in computed tomography (CT) angiography.

	PAOI	
	Spearman's correlation coefficient (*ρ*)	P Value
LA Long Diameter, cm	-0.205	0.152
LA Short Diameter, cm	-0.380	**0.006**
LA Height, cm	-0.483	**<0.001**
LA Area, cm^2^	-0.449	**0.001**
LA Volume, cm^3^	-0.472	**0.001**

RA Long Diameter, cm	-00.089	0.538
RA Short Diameter, cm	.334	**0.018**
RA Height, cm	-0.144	0.320
RA Area, cm^2^	0.041	0.776
RA Volume, cm^3^	-0.015	0.916

RA to LA Long Diameter Ratio	0.05	0.68
RA to LA Short Diameter Ratio	0.50	**<0.01**
RA to LA Height ratio	0.25	0.08
RA to LA Area Ratio	0.44	**<0.01**
RA to LA Volume ratio	0.39	**<0.01**

Reflux Grade, n	-0.033	0.822

*Abbreviations*: LA: left atrium; PAOI: pulmonary artery obstruction index; RA: right atrium.

**Table 3:** ROC curve analysis of PAOI and different RA and LA measurements.

					90%	Sensitivity
	AUC	95%	CI	P Value	Cut-off	Specificity
LA Long Diameter, cm	0.634	0.512 -	0.756	**0.035**	5.32	75.0
LA Short Diameter, cm	0.665	0.548 -	0.782	**0.009**	2.83	77.8
LA Height, cm	0.426	0.303 -	0.548	0.241	3.95	94.4
LA Area, cm^2^	0.675	0.554 -	0.796	**0.006**	13.06	69.4
LA Volume, cm^3^	0.609	0.487 -	0.731	0.086	33.09	80.6

RA Long Diameter, cm	0.693	0.577 -	0.809	**0.002**	4.07	55.6
RA Short Diameter, cm	0.624	0.502 -	0.747	**0.050**	4.14	83.3
RA Height, cm	0.550	0.428 -	0.672	0.431	5.75	88.9
RA Area, cm^2^	0.724	0.609 -	0.839	**<0.001**	15.61	58.3
RA Volume, cm^3^	0.709	0.594 -	0.824	**0.001**	62.83	58.3

RA to LA Long Diameter Ratio	0.610	0.489 -	0.731	0.083	0.60	83.3
RA to LA Short Diameter Ratio	0.432	0.309 -	0.554	0.282	0.98	94.4
RA to LA Height Ratio	0.572	0.451 -	0.693	0.259	1.08	88.9
RA to LA Area Ratio	0.553	0.430 -	0.676	0.406	0.69	88.9
RA to LA Volume Ratio	0.548	0.426 -	00.670	0.446	0.78	100

*Abbreviations*: AUC: area under the curve; CI: confidence interval; cm: centimeter; LA: left atrium; PAOI: pulmonary artery obstruction index; RA: right atrium.

## Data Availability

The SPSS compatible version of data used to support the findings of this study was supplied by Cardiovascular Research Center of Shahid Beheshti University of Medical Sciences, under license, and so cannot be made freely available. Requests for access to these data should be made to Dr. Isa Khaheshi (isa.khaheshi@gmail.com).
